# Diagnostic angiography for identification and management of late vascular injuries in war-related traumatic peripheral vascular injuries: A retrospective cohort study

**DOI:** 10.1371/journal.pone.0319761

**Published:** 2025-03-18

**Authors:** Efrat Keren Gilat, Boris Khaitovitch, Yiftach Barash, Noam Tau, Eli Konen, Moshe Halak, Daniel Silverberg, Barak Raguan, Vera Sorin, Daniel Raskin

**Affiliations:** 1 Division of Diagnostic Imaging, Sheba Medical Center, Ramat Gan, Israel; 2 Faculty of Medicine, Tel Aviv University, Tel Aviv, Israel; 3 Department of Vascular Surgery, Sheba Medical Center, Ramat Gan, Israel; 4 Trauma Unit, Division of General Surgery, Sheba Medical Center, Ramat Gan, Israel; The Fourth Affiliated Hospital Zhejiang University School of Medicine, CHINA

## Abstract

**Purpose:**

One of the feared complications of war-related peripheral vascular injury is the development of delayed hemorrhage. This study describes our experience with an innovative protocol of surveillance diagnostic angiography to detect occult late vascular complications in an effort to prevent delayed hemorrhage.

**Materials and Methods:**

This retrospective cohort study was conducted at a single level one trauma center, reviewing patients with war-related peripheral vascular injuries caused by penetrating trauma from October 7th, 2023, to January 21st, 2024. Data collected included patient demographics, primary injury characteristics, associated complications, incidence of late vascular injuries (either symptomatic or occult), means of diagnosis, treatment strategies and outcomes.

**Results:**

The cohort included 41 patients with war-related peripheral vascular injuries affecting 51 limbs. All patients were male (100%) with a median age of 25 years, the majority being soldiers (85%). 24 occurrences of late vascular injuries were observed in 22 (43%) out of 51 limbs (100%). Half were symptomatic, with delayed hemorrhage occurring in 5 limbs in total (10%), and half were asymptomatic. A total of 17 surveillance diagnostic angiographies were performed with the sole indication of identifying occult late vascular injuries in asymptomatic patients, of which 4 (24%) were positive for findings. Five additional diagnostic angiographies were performed to assess late injuries discovered incidentally on imaging studies that were performed for other indications, and all were positive for late vascular injuries. Of all late vascular injuries, a total of 83% required subsequent treatment.

**Conclusions:**

Late vascular injuries are a potentially lethal complication of war-related peripheral vascular injury. Aggressive surveillance with diagnostic angiography prior to discharge from a high intensity care unit can detect asymptomatic late vascular injuries, the treatment of which may prevent life-threatening hemorrhage.

## Introduction

Penetrating trauma, predominantly from high-energy gunshot wounds and blast injuries, is the primary cause of vascular injuries among young soldiers aged 20-40 years [1–3]. On the battlefield, external hemorrhage control and prompt medical evacuation play vital roles in reducing mortality and preventing irreversible damage from limb ischemia [1,4–6]. However, management of these injuries extends beyond the immediate battlefield scenario.

An oft-neglected yet well-known complication became appreciated: delayed hemorrhage from injured extremities [7]. This is often due to disruption of the vascular anastomosis, which in turn is often related to the surrounding injury to the soft tissue, or to underappreciated damage to the remaining blood vessel [7–9].

This study aims to share insights regarding the role of interventional radiology (IR) following recent experience during mass casualty military conflict, focusing on its effectiveness in detecting and treating late vascular injuries. This includes assessing the yield of an interval pre-discharge diagnostic angiography. We hypothesize that the use of surveillance angiography to detect these injuries might decrease the incidence of delayed, life-threatening hemorrhage.

## Materials and methods

This single-center retrospective cohort study was approved by an institutional review board (IRB), the Sheba IRB-Helsinki Committee. Informed consent was waived due to the retrospective nature of this study.

Data were accessed for research purposes between November 23^rd^, 2023, and January 21^st^, 2024. Authors had access to information that could identify individual participants during data collection.

### Patient selection

This study is a retrospective analysis of patient records from a level one trauma center, covering the time period from October 7th, 2023, to January 21st, 2024. Included are all consecutive patients who sustained peripheral vascular injuries due to penetrating trauma in military combat, including those directly evacuated from the battlefield with primary injuries and those transferred from other hospitals following initial intervention. Peripheral vascular injury was defined as injuries identified intraoperatively or through findings on computed tomographic angiography (CTA) or other imaging modalities, which include complete or partial transection, occlusion, extravasation, and arteriovenous fistula (AVF). Peripheral injuries included extremity and junctional vessels.

During the study period, overall, 247 patients (242 males and 5 females, age range 18-53 years, median age 24 years) who sustained combat injuries were admitted to our medical center. Of those, 41 patients who sustained war-related penetrating trauma to the extremities, and either presented with or later developed peripheral vascular injury, were included in the analysis. The number of patients treated for their initial injury at a different hospital was 23 (56%) and they were either transferred for further care at our hospital, admitted to our hospital’s rehabilitation center, or turned seeking care for either follow-up or new symptoms.

### Patient assessment and management

We retrospectively collected data from the hospital’s electronic medical records system (Chameleon, Elad Group, Israel), Radiology Information System (RIS), and the Picture Archiving and Communication System (PACS) (Carestream, Philips Healthcare, Netherlands). For each patient, the following data was collected: demographic information, including age, gender and association to the armed forces; details on the initial injury, such as mechanism of injury, imaging findings, the type of injury as defined by intraoperative and/or imaging findings, and management strategies both in the field (tourniquet application) and in the hospital setting (surgical, endovascular, or conservative management). In our facility surgical repair of vascular injury is always followed by completion angiography, all intraoperative findings including the findings at completion angiography were documented. Further data included early and late complications such as infection, rhabdomyolysis and the presence of late vascular injury. Late vascular injuries were defined as injuries not initially presenton the index imaging (either CTA or intra-operative angiography).

Patients were followed up for at least 30 days. The correlation between several of the aforementioned factors and the presence of late vascular injuries was assessed.

Patients underwent an endovascular procedure either due to relevant symptoms or as a part of a pre-discharge angiographic evaluation for asymptomatic patients. Our pre-discharge surveillance angiography protocol was determined to include patients with peripheral vascular injuries, after amputation or vascular intervention. The vascular interventional radiologists’ role in the management of both primary and late vascular injuries was assessed.

### Imaging technique

In the CTA scans performed, patients were scanned in one of two 64-slices CT scanners: Revolution EVO and GE CT 750 HD (GE Medical Systems, Milwaukee, WI) using the same dedicated protocol: Scan area from the level of the clavicles to the toes with the region of interest placed at the descending aorta, on the level of the carina with a threshold of 180 Hounsfield Units. Intravenous contrast injection of 120 ml contrast material, with a rate of 4.0 ml/sec. Scans were performed in an arterial phase for the chest, abdomen, and legs with arms included in the scan field (20 seconds post-threshold), and a venous-portal phase of the abdomen (80 seconds post-threshold).

Endovascular procedures were conducted in two dedicated angiography suites (Artis Q, Siemens Healthineers, Forchheim, Germany; Siemens ARTIS pheno, Siemens Healthineers, Erlangen, Germany) by a team of staff vascular interventional radiologists.

### Statistical analysis

Descriptive statistics were calculated with Microsoft Excel (Microsoft, Redmond, WA). Results are presented as ranges and medians. Categorical data were presented as frequencies and percentages and analyzed using Pearson’s chi-square or Fisher exact test. Numerical data were analyzed using Mann-Whitney U Test. A p value of <  0.05 was considered statistically significant.

## Results

Patient demographic information and data on the mechanism of injury, location, and concomitant fractures are presented in Table 1.

**Table 1:** Patient demographics and primary injury characteristics (n = 41).

Patient demographics		
Total number of Patient	**41**	
Male	**41** (100%)	
Soldiers or Armed Forces	35 (85%)	
Civilians	6 (15%)	
Age (range, median)	20-52, 25	
Penetrating injury characteristics		
Total number of injuries (limbs)	**51**	
Mechanism	Gunshot	29 (57%)
	Explosives	22 (43%)
Gross location	Upper extremity	14 (27%)
	Lower Extremity	34 (67%)
	Pelvis/gluteal	3 (6%)
Concomitant fracture	27 (53%)	

Results are presented as number (percent).

Out of 51 limbs involved with documented vascular injury, there were 48 limbs with obvious or suspected peripheral vascular injuries at presentation, and 3 limbs not initially suspected to have vascular injury, which were diagnosed with vascular injury later on. Of the primarily suspected peripheral vascular injuries, 32/48 (67%) were managed in the field by tourniquet application, 11/48 (23%) had no tourniquet upon arrival to the hospital, notably some of them owing to the location of the injury (scapula, pelvis/buttocks), and 5/48 (10%) had no proper documentation of tourniquet application. Of the cases where a tourniquet was applied, documentation of duration was also lacking in 15/32 cases (46%).

### Primary vascular injury

**Fig 1:**
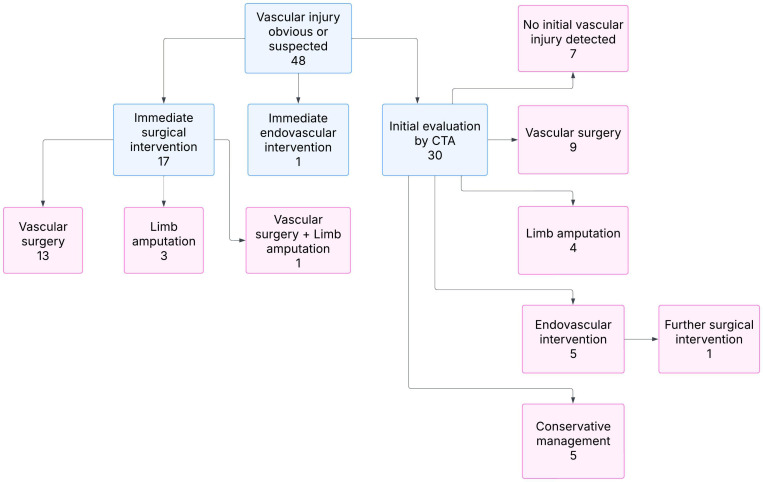
Initial injury management. Flow chart of initial management of suspected vascular injuries, the flow chart includes all extremity injuries following penetrating trauma that were evaluated for obvious or suspected peripheral vascular injury upon arrival.

Initial management upon arrival at the hospital is depicted in Fig 1. Vascular injuries that warranted immediate surgical exploration at presentation were first assessed intraoperatively and treated accordingly. The rest were first assessed by CTA prior to the determination of the therapeutic strategy. Table 2 details characteristics of primary peripheral vascular injuries.

**Table 2:** Primary vascular injuries characteristics (n = 41).

Parameters			n (%)
Type of vascular injury (n, %)	Arterial transection		27 (66%)
	AVF		5 (12%)
	PSA		1 (2%)
	Mangled extremity		8 (20%)
Artery involved (n, %)	SFA		14 (34%)
	PTA		6 (15%)
	Popliteal a.		3 (7%)
	Peroneal a		3 (7%)
	ATA		3 (7%)
	PFA		1 (2%)
	TPT		1 (2%)
	Gluteal or another internal iliac a. branch		3 (7%)
	Axillary a.		2 (5%)
	Brachial a.		5 (12%)
	Ulnar a.		2 (5%)
	Radial a.		2 (5%)
Mechanism of primary injury	Gunshot		22 (54%)
	Explosives		19 (46%)
Management	**Surgical**	**Total**	**31 (76%)**
		Primary vessel repair	9/31 (29%)
		SVG bypass	12/31 (39%)
		Ligation of bleeding vessel	5/31 (16%)
		Thrombectomy	3/31 (10%)
		Limb amputation	8/31 (26%)
	**Endovascular**	**Total**	**6 (15%)**
		Embolization	4/6 (67%)
		Stent graft	1/6 (17%)
		Diagnostic	1/6 (17%)
	**Conservative**	**Total**	**5 (12%)**
Associated venous injury i.e., Venous transection/laceration	5 (12%)		

Results are presented as number (percent) of total patient cohort, unless specified otherwise. AVF = arteriovenous fistula, ATA=anterior tibial artery, PFA =  profunda femoris artery, PSA=pseudoaneurysm, PTA=posterior tibial artery, SFA=superficial femoral artery, SVG=saphenous vein graft, TPT=tibioperoneal trunk

### Late vascular injury

**Fig 2:**
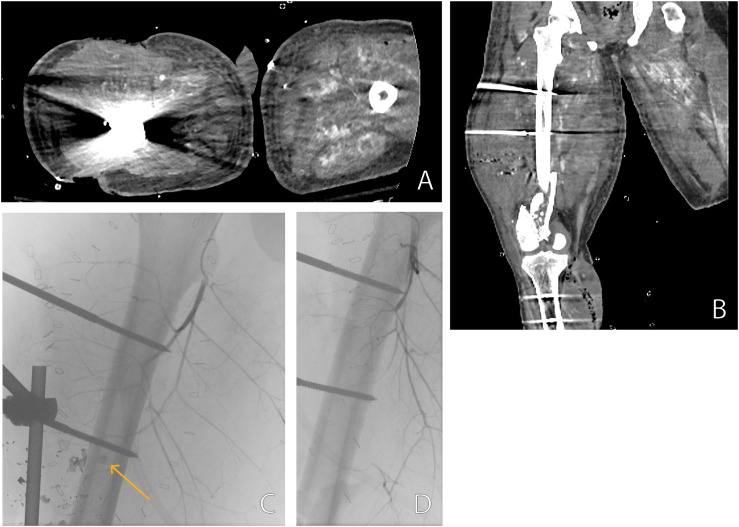
(A-D). Exemplary case of late vascular injury. Axial (A) and coronal (B) CTA of a 22 years old soldier who suffered multiple gunshot wounds, performed one month after the initial injury. Artifacts from external fixation of the femur and signs of rhabdomyolysis such as mild fiber-like enhancement after contrast administration and skeletal muscle calcification are visible, obscuring vascular structures and possible pathologies. Image C shows a same day angiography of the same limb, revealing a pseudoaneurysm of a PFA branch (arrow). The leading artery was embolized with Concerto Detachable Coil, image D shows the post procedure resolution of the pseudoaneurysm.

During the initial few months after the war outbreak, we observed numerous cases of late vascular injuries. Initially, symptomatic patients underwent CTA for peripheral vasculature evaluation. However, in one case of a patient after bypass surgery who also developed severe rhabdomyolysis, CTA proved irrelevant for assessing the vasculature in his lower limb owing to several factors as shown in Fig 2. Diagnostic angiography in this patient which was performed on the same day revealed a pseudoaneurysm (PSA) in the first perforating branch of the profunda femoris artery (PFA), which was embolized by coiling. This observation prompted the establishment of a dedicated facility protocol, dictating that patients with peripheral vascular injuries caused by penetrating trauma, post-amputation or vascular intervention, undergo diagnostic angiography before discharge with the aim of identifying and treating late injuries.

Follow-up time ranged from 30 days to 128 days post-injury, with a median of 68 days. In this follow-up period, 24 instances of late vascular injury were observed, involving 22 extremities, which makes 43% of the cohort. Characteristics of these either delayed or missed peripheral vascular injuries, including what led to their diagnosis, time lag to diagnosis and following management strategies, are detailed in Table 3.

**Table 3:** Late vascular injury characteristics (n = 24).

Characteristics		n (%)	
Cause of diagnosis	**Symptomatic**	**Total**	**12 (50%)**
		Overt Hemorrhage	6/12 (50%)
		Limb swelling/compartment syndrome	4/12 (33%)
		Signs of limb ischemia	1/12 (8%)
		Paresthesia	1/12 (8%)
	**Asymptomatic (incidental)**	**Total**	**12 (50%)**
		Routine diagnostic angiography	4/12 (33%)
		US/CT for other indication	8/12 (67%)
Time from initial trauma to diagnosis	≤7 days	5 (21%)	
	8-30 days	10 (42%)	
	>30 days	9 (37%)	
Type of vascular injury	PSA	13 (54%)	
	AVF	7 (29%)	
	Vessel occlusion	3 (13%)	
	Active bleeding	8 (33%)	
Artery involved	SFA	4 (17%)	
	PTA	5 (21%)	
	Popliteal a.	3 (13%)	
	Peroneal a	1 (4%)	
	ATA	2 (8%)	
	PFA	2 (8%)	
	Gluteal or another internal iliac a. branch	2 (8%)	
	Axillary a.	1 (4%)	
	Brachial a.	2 (8%)	
	Ulnar a.	1 (4%)	
	Radial a.	2 (8%)	
Mechanism of primary injury	Gunshot	16 (67%)	
	Explosion	8 (33%)	
Associated complications	Amputation	9 (38%)	
	Wound or deep tissue infection	15 (63%)	
	Rhabdomyolysis	14 (58%)	
	Concomitant fracture	12 (50%)	
Management	**Surgical**	**Total**	**8 (33%)**
		Primary repair	3/8 (38%)
		SVG Bypass	3/8 (38%)
		Ligation of bleeding vessel	2/8 (25%)
	**Endovascular**	**Total**	**12 (50%)**
		Stent graft	3/12 (25%)
		Embolization	8/12 (67%)
		Thrombosis aspiration	1/12 (8%)
	**Conservative**	**Total**	**4 (17%)**

Results are presented as number (percent) of total, unless specified otherwise. AVF = arteriovenous fistula, ATA=anterior tibial artery, CT = computed tomography, PFA =  profunda femoris artery, PSA=pseudoaneurysm, PTA=posterior tibial artery, SFA=superficial femoral artery, SVG=saphenous vein graft, TPT=tibioperoneal trunk, US = ultrasonography

A higher rate of delayed or missed vascular injuries was found in cases where no preoperative CT imaging was obtained before the index operation (10/17, 59%), than those that were initially evaluated by imaging at presentation (11/30, 37%), but the difference was not statistically significant (p = 0.22).

There was no significant difference in the time between initial injury and diagnosis of late vascular injury between symptomatic and non-symptomatic patients, with a median of 25 days (range 1-104) for symptomatic and 26.5 days (range 1-91) for non-symptomatic patients. Injuries following gunshot wounds were more delayed in diagnosis, with a range of 1-104 days following initial injury and a median time to diagnosis of 33.5 days. Missed or delayed injuries following blast injury, on the other hand, were diagnosed earlier, with a range of 1-81 days and a median of 8 days. There was no discernable difference between different initial management strategies and the time lag to diagnosis, with a median time of 23.5 days (range 1-81 days) and 25 days (range 1-91 days) for injuries first managed by immediate surgery and injuries first evaluated by CTA, respectively.

Of the twelve incidentally discovered late injuries in asymptomatic patients, ten (10/12, 83%) required further intervention.

### The role of interventional radiology

Overall, 31 endovascular procedures were performed on 26 limbs in 23 patients. Indications for endovascular procedures and the pathologies found are summarized in Table 4.

**Table 4:** Endovascular procedures performed during study period: indications and number of pathologic findings.

		Number of procedures (%)	Pathological findings (%)
Total		31	16/31 (52%)
Indication			
	Symptomatic	9/31 (29%)	7/9 (78%)
	Asymptomatic	22/31 (71%)	9/22 (41%)
Findings		Extravasation	4/16 (25%)
		PSA	9/16 (56%)
		AVF	7/16 (43%)
		Vessel occlusion	2/16 (13%)

Results are presented as number (percent) of total, unless specified otherwise. Abbreviations: AVF = arteriovenous fistula, PSA=pseudoaneurysm

For patients with symptoms warranting endovascular workup and treatment, symptoms included overt hemorrhage (n = 4), limb swelling (n = 1), signs of limb ischemia (n = 1), paresthesia (n = 1), and unexplained low hemoglobin levels (n = 2).

We documented three cases of severe hemorrhage occurring after limb amputation – 16-, 24- and 104-days post-amputation. These patients all had concomitant wound infection and rhabdomyolysis. All three were successfully managed via endovascular embolization. Three further cases of overt hemorrhage included breakdown of anastomosis, occurring 23-, 26- and 30-days post primary repair, notably two of them in the same patient. Both patients had concomitant infection and rhabdomyolysis. Two cases were treated surgically, and the case of recurrence of anastomosis breakdown was managed with a combination of surgery and endovascular embolization.

Diagnostic angiographies were performed in patients without presenting symptoms either (1) prior to patient discharge per the newly implemented facility protocol (17/22, 77%), four of which were positive for incidental pathological findings (4/17, 24%) including three PSAs and one case of ulnar artery occlusion, or (2) due to incidental findings discovered on other imaging modalities performed for unrelated indications (5/22, 23%), all of which were positive for findings as expected (5/5, 100%), which included seven PSAs and two AVFs.

Out of the total 16 procedures with pathological findings, 12 (75%) cases were treated intra-procedurally as detailed in Table 3. Stent graft placement was utilized for the treatment of PSAs, thrombus aspiration for vessel occlusion, and either coiling or a combination of coiling and liquid embolics for the treatment of both PSAs and AVFs. In three cases (19%), conservative management was opted for, and patients were instructed to return for follow-up examinations. One case (6%) was further managed by vascular surgery, consisting of primary repair of the brachial artery.

Of the nine pathologies incidentally found on angiography, seven (7/9, 78%) underwent further endovascular treatment and two (2/9, 22%) were managed conservatively. Notably, none had further symptomatic or incidentally found vascular injury during the follow up period.

No IR procedure-related complications such as entry site PSAs or contrast-related acute kidney injury were observed (0%).

### Other associated complications

Twenty-seven patients from the entire cohort (66%) suffered severe wound and soft tissue infections, involving 29 limbs. Deep tissue biopsies and superficial swab cultures isolated unusual multibacterial and fungal infections (S1 Table). Fifteen (52%) out of 29 infected limbs developed late vascular injury, as opposed to nine (41%) out of 22 non-infected limbs, a difference which was not statistically significant (p = 0.23). Notably, three out of fifteen cases of late vascular injuries associated with infected limbs were discovered less than a week after the initial injury and therefore are not necessarily related to the infection.

Out of 25 cases with rhabdomyolysis, 14 (56%) had delayed or missed vascular injuries as opposed to ten (38%) out of 26 cases without rhabdomyolysis, the difference was not statistically significant (p = 0.33).

Missed or delayed vascular injury was associated with longer hospitalization durations, with a median of 46 days (range 3-128 days) compared to 25 days (range 3-86 days) in cases without secondary vascular injury detected during the observation period (p = 0.04). There were no cases of death.

## Discussion

Management of trauma patients, particularly in combat-related trauma, is inherently complex due to the diverse and severe nature of their injuries. This complexity often obscures the entire picture, posing a significant challenge in accurately diagnosing the full range of injuries. In our cohort, instances of delayed or missed vascular injuries were observed in 43% of involved limbs, in both symptomatic and asymptomatic patients. Delayed hemorrhage occurred in 5 limbs (5/51, 10%), with an extra case of recurring anastomosis breakdown in one of them. A locally developed pre-discharge angiographic surveillance protocol helped identify and treat delayed or missed vascular injury in 24% of procedures, but additional incidental late injuries were discovered through other modalities. Out of the incidentally discovered injuries, 83% required further treatment. Endovascular intervention was the most often used management strategy for secondary injuries, applied in half of the cases.

This single-center study, drawing from experience during the recent conflict [10], emphasizes the importance of collaborative efforts and a multi-disciplinary approach in management of such complex injuries.

In the acute setting, the initial management of vascular injuries is led by trauma and vascular surgeons, and typically involves surgical intervention [3,11–18], with interventional radiologists primarily counseling on the interpretation of vascular findings on CTA, the main diagnostic tool, offering a rapid and highly sensitive method for diagnosing arterial injuries [1,19–24]. Endovascular management, such as arterial embolization for hemorrhage control in branch vessel injuries, may be used depending on factors such as injury location, associated injuries, patient hemodynamics, and available expertise [14,25–28]. This was the case in our experience as well, where endovascular treatment was reserved for a few select cases of primary injuries.

However, when dealing with delayed or missed vascular injury, endovascular management has emerged as a more dominant player. The likelihood of missed vascular injuries or delayed complications increases in multi-trauma or mass casualty situations, particularly in cases of traumatic injuries from military ammunitions [11,15,29–31]. Complications like hemorrhage, PSAs, AVFs, and occlusion can be diagnosed even months after the initial injury [29,31]. When the assessment of these injuries on CTA is ambiguous or inconclusive due to artifacts from foreign objects, such as external and internal fixation or metal fragments and shrapnels, or confounded by imaging characteristics of rhabdomyolysis [32], angiography becomes critical for both diagnosis and treatment [1,33]. This dual role is particularly beneficial in managing complex trauma patients and has previously been shown to be achievable and effective in such cases [17,27,34–37].

In our experience, similar to previous experience from combat settings, vascular extremity injuries have a small but substantial risk for delayed hemorrhage. The theories to explain this include (1) missed diagnosis (2) evolution of the injury due to unappreciated or undetected tissue damage from the initial injury or (3) new injuries due to tissue infection and ischemia [7–9]. This risk manifested in several cases of severe hemorrhage after surgical repair which along with incidentally found vascular injuries prompted us to develop a local protocol for the detection of these late injuries.

Previous studies have recommended close monitoring and arteriography to evaluate patients with potential vascular injury, even when clinical suspicion is low [15,31,34]. Fox et al. specifically evaluated vascular injury management among wartime evacuees, employing interval arteriography for a subset of patients with confirmed or strongly suspected vascular injury. The most common indications were the mechanism of injury, abnormal examination, operative planning, or evaluation of a repair. This approach enabled the detection of late injuries, some of which required subsequent intervention. Angiography performed solely on the basis of the mechanism of injury were positive 25% of the time [15]. Similarly, our results show non-negligible rates of late vascular injury, the majority of which required intervention. Notably, half of these cases were asymptomatic, with injuries detected incidentally, and most treated by endovascular approach. Implementation of a new facility protocol, conducting routine pre-discharge diagnostic angiography in asymptomatic patients with peripheral vascular injuries from penetrating extremity trauma, revealed vascular findings in 24% of cases, the majority of which were deemed significant enough to be intervened upon. While some occult lesions may resolve spontaneously, there is no sure way to assess which discovered incidental injury might develop into a limb-threatening or life-threatening one. Of course, our protocol aims to identify and treat those injuries with potential clinical significance in order to prevent possible complications, which we saw none of in the remaining follow up time in those patients for whom intervention was opted for in management of their incidentally found injuries. Consequently, we advocate for routine angiographic assessment in certain asymptomatic patients following penetrating trauma, especially when caused by gunshot or blast wounds, in order to facilitate timely diagnosis and therapeutic intervention. While not statistically significant, the trend towards increased rates of late vascular injuries associated with infection or rhabdomyolysis suggests an advisable high index of suspicion in patients with these conditions.

### Limitations

The study’s retrospective nature and the modest size of our cohort present inherent limitations, particularly in terms of the generalizability and robustness of the findings. These factors may constrain our ability to draw broad conclusions from statistical analyses, hence the descriptive nature of our report. Despite these limitations, we believe our study still offers valuable insights into the trends and nature of secondary vascular injuries. Moreover, the incidental discovery of vascular injuries in cases not initially suspected to have such injuries highlights an area of underestimation in the wider population of war casualties. This underscores the need for a high index of suspicion for vascular injuries in penetrating trauma patients, particularly those with extremity injuries, and reinforces the importance of comprehensive diagnostic approaches in trauma care.

Another factor limiting the interpretation of our findings is the definition of late vascular injuries. We used the term ‘late’ to group together both delayed and missed injuries, since many times those were de-facto indistinguishable due to the time frame or lack of initial imaging. The nature of delayed and missed injuries is obviously different, as one is postulated to occur de-novo after the initial injury due to tissue damage and other complications, and the second thought to be caused by the same mechanism as the initial injury but not detected at initial evaluation, respectively. However, we saw it fit to include any late vascular injury as the presence of both types emphasizes the utility of angiography, and also as not to underestimate the occurrence of delayed injuries.

## Conclusions

This study highlights the important role of angiography in the early detection and management of late peripheral vascular injuries in trauma patients following penetrating extremity injuries, marking a significant paradigm shift in the application of angiography in trauma care. While prior studies have documented vascular injuries in combat settings, our study highlights the possible benefit of pre-discharge angiographic evaluation in asymptomatic patients. By implementing a facility protocol for pre-discharge angiographic evaluations, we were able to identify vascular injuries that might have otherwise been overlooked. Our findings advocate for the refinement of trauma care protocols to integrate these insights, focusing on early identification and proactive intervention for delayed or missed vascular complications in order to prevent potential morbidity and mortality, especially in the context of mass casualty military conflict.

## Supporting Information

S1 Table Deep tissue biopsies and superficial swab cultures isolated bacterial and fungal pathogens.
Results are presented as number (percent) of total. Including only pathogens isolated in over 10% of cases.
(DOCX)
