# Supplementary material for: Diagnostic angiography for identification and management of late vascular injuries in war-related traumatic peripheral vascular injuries: A retrospective cohort study
Source: PLoS One. 2025 Mar 18;20(3):e0319761. doi: 10.1371/journal.pone.0319761 (PMC11918411; doi:10.1371/journal.pone.0319761)
Supplement: S1 Table — Results are presented as number (percent) of total. Including only pathogens isolated in over 10% of cases. (DOCX) [file pone.0319761.s001.docx]

Supplementary Table 1: Deep tissue biopsies and superficial swab cultures isolated bacterial and fungal pathogens.

|  | | | |  |  |
| --- | --- | --- | --- | --- | --- |
| Total number of infected limbs (n) | | | 29 |  |  |
|  | **Isolated pathogen** | **n , %** | | | |
| Bacterial (n, %) | Enterococci (most commonly E. Faecium, E. Faecalis) | 11, 41% | | | |
|  | Enterobacter Cloacae | 9, 33% | | | |
|  | Staphylococcus Epidermidis | 6, 22% | | | |
|  | Pseudomonas Aeruginosa | 6, 22% | | | |
|  | Stenotrophomonas Maltophilia | 6, 22% | | | |
|  |  |  | | | |
| Fungal (n, %) | Fusarium species | 5, 19% | | | |
|  | Aspergillus species | 4, 15% | | | |
|  | Candida Albicans | 3, 11% | | | |

Results are presented as number (percent) of total. Including only pathogens isolated in over 10% of cases.
